# Growth performance, meat yield and blood lipid profile of broiler and Sonali chickens

**DOI:** 10.1016/j.vas.2022.100272

**Published:** 2022-11-23

**Authors:** Mohammad Aminul Islam, Ainul Haque, Masahide Nishibori

**Affiliations:** a Department of Dairy and Poultry Science, Bangabandhu Sheikh Mujibur Rahman Agricultural University, Gazipur, 1706, Bangladesh; b Lab of Animal Genetics, Graduate School of Integrated Sciences for Life, Hiroshima University, 1-4-4, Kagamiyama, Higashi-Hiroshima, 739-8528, Japan

**Keywords:** Chicken type, Cholesterol, Growth, Meat, Production cost, Net profit

## Abstract

A total of 14,200, day-old broiler chicks were allotted into two batches (B_1_=Winter and B_2_ = Summer) with 6 replicates each for 30 days, and 16,000, day-old *Sonali* chicks were allotted into 2 batches with 4 replicates each for 60 days to assess the growth performance, meat yield, and lipid profiles of the blood of chickens. Broiler chickens showed significantly higher body weight, feed intake, and lower FCR and production cost with a tendency to increase mortality compared with *Sonali* chickens. However, net profit tended to be higher in *Sonali* chickens compared to broiler chickens. The higher meat yield traits were observed in the broiler chicken compared with the *Sonali* chicken (*p*<0.001). Lipid profile did not differ (*p*>0.05) between chicken types. However, lipid profiles tended to be higher in broiler chicken than in *Sonali* chicken, except for the low-density lipoprotein (LDL). Growth performance, meat yield traits, and lipid profiles did not differ (*p*>0.05) between batches, except for the dressing percentage. Dressing (%) was higher in B_1_ than in B_2_ (*p*<0.05). No interaction between batch and chicken type was found in the growth performance, meat yield, and blood lipid profile of chickens. Therefore, broiler chickens performed better than *Sonali* chickens in terms of growth and meat yield traits. Nevertheless, *Sonali* chickens tended to perform better than broilers in terms of consumer preference, net profit, and lowering total cholesterol and triglyceride. However, more studies are needed to confirm the present findings and make the suggestion to use a suitable chicken type for meat production.

## Highlights

• Comparative growth, meat yield and lipid profiles of broiler and *Sonali* chickens were investigated.
• Broiler chickens performed better than *Sonali* chickens in terms of growth and meat yield traits.
• *Sonali* chicken tended to reduce serum cholesterol and increase net profit (*p*>0.05).
• *Sonali* chicken was superior to broiler in terms of preference, net profit and serum cholesterol content.

## Graphical abstract

**Figure:**
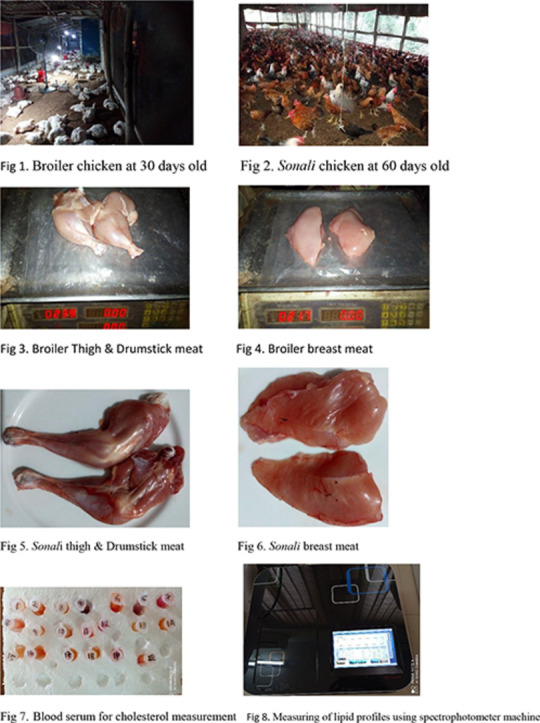

## 1 Introduction

Chicken meat is one of the cheapest and most available sources of animal protein, preferred by all classes of people irrespective of religion and age. Poultry farming provides meat and egg essential for humans (Regar et al., 2019). Poultry contributes about 22–27% of the total animal protein from different animal sources (Islam et al., 2019). Poultry is the most important and emerging sector of Agriculture in Bangladesh. Over the years, the demand for poultry products (meat and eggs) in Bangladesh has grown significantly; the consumption (per capita per year) of poultry meat and egg in 2019 was 8.5 kg and 104 pieces, respectively. However, to meet the growing domestic demand for meat and eggs, there is a need to increase poultry production (EKNB, 2020).

As per the Department of Livestock Services (DLS) report (2020), the total poultry population of Bangladesh is about 356.318 million of which chickens are 296.602 million. An adult person needs 120 g of meat per day and 104 eggs annually. Therefore, the availability of meat (g/day/person) and egg (per person per year) is 126.20 and 104.23, respectively. The annual meat and egg production in Bangladesh is 7.67 million metric tons and 17,364.30 million, respectively which can satisfy the requirement of the people (DLS, 2020). The poultry industry is growing rapidly and contributes 1.5-1.6 percent to the country's GDP, involving at least 6.0 million people in this sector (Karmoker, 2022). In the poultry sector, broiler meat is produced within the shortest possible period with a minimum investment having maximum profit (Whitehead, 2002).

Poultry meat and eggs are easily digestible and minimize the risks of blood pressure, heart diseases, and prevent cancer in human beings (Mazmanyan, 2021). Broiler meat is very popular with consumers regardless of age and religion because of its availability, digestibility, and lower price. It is also advantageous because of its rapid growth, better feed conversion efficiency, and meat quality, especially for breast meat or lean meat (Amin et al., 2013).

*Sonali* is a crossbred of Rhode Island Red (RIR) x Fayoumi with a phenotypic appearance similar to a local chicken called ‘*Sonali*’ are well adapted to tropical climates that require less care and attention to rear compared with broiler chickens (Saleque & Saha, 2013). At present, *Sonali* provides about 30% of the total required chicken meat in Bangladesh (Huque et al., 2011). Consumers prefer *Sonali* chicken meat over regular broiler meat and it looks like indigenous chicken meat. In 2018, the percentage of preference for *Sonali* chicken and around 20% of total poultry meat reached 23% in 2019 (EKNB, 2020).

Although *Sonali* crossbred is popular with the farmers but the problem is a slower growth rate. It takes more than 60 days to get a marketable live weight (1.00 kg). The rearing of *Sonali* birds is more profitable than commercial broilers. Practically it has been found that farmers earned more returns from *Sonali* chicken farming than from commercial broiler farming. Because of the higher prices of *Sonali* birds compared to the commercial broilers (Azharul et al., 2005; Jahan et al., 2021). In addition, the price fluctuation of *Sonali* chickens is lesser than commercial broilers, because consumers prefer *Sonali* chickens to broilers (Uddin et al., 2015). Nowadays, consumers are so conscious of the carcass quality and lipid profile content of the bird (Jahan et al., 2015). In the case of a broiler, fat is deposited into the abdomen which deteriorates the meat quality and increases the cholesterol level in the meat which is so harmful to the body of human beings (Tohala, 2010). On the other hand, *Sonali* chickens have no abdominal fat which is why *Sonali* chickens produce quality meat. Therefore, consumers prefer *Sonali* chicken meat more than broiler chicken meat.

Considering the above points, the present study was planned to assess the growth performance, meat yield, and lipid profiles of broiler and *Sonali* chickens to identify a potential chicken type for producing quality, preferable and cost-effective chicken meat.

## 2 Materials and methods

### 2.1 Experimental site

The experiment was carried out at different commercial broiler and *Sonali* chicken farms located at Subarnachar Upazila, Noakhali district, and the laboratory of the Department of Dairy and Poultry Science, Faculty of Veterinary Medicine and Animal Science, Bangabandhu Sheikh Mujibur Rahman Agricultural University, Bangladesh

### 2.2 Preparation of poultry house and diet

The house was prepared before receiving the day-old chicks in the house. The diets were prepared by ‘Nahar Poultry Feed’ and ‘Teer Poultry Feed’ Mill for broiler and *Sonali* chickens as per their requirements, respectively.

### 2.3 Feeding trial

A total of 14,200, day-old broiler chicks were allotted into two batches (B_1_ = Winter and B_2_ = Summer) with 6 replicates each for 30 days, and 16,000, day-old *Sonali* chicks were allotted into 2 batches with 4 replicates each for 60 days (Table 1). The birds were reared on a littered floor management system. The broiler chicks were fed a starter diet containing 22% CP and 3035 Kcal ME/kg (1–10 days) and a finisher diet containing 21% CP and 3150 Kcal ME/kg (11–30 days) (Table 2). The *Sonali* chicks were fed a starter diet containing 19.50% CP and 2950 Kcal ME/kg (1–30 days) and a finisher diet containing 19.00% CP and 3000 Kcal ME/kg (31–60 days) (Table 2). Clean and fresh water was provided *ad-libitum* to the birds during the experimental period. The standard management practices as per the standard of the breeder were provided to the birds during the investigation.

**Table 1:** Layout for the determination of the growth performance of the broiler and *Sonali* chickens.

Chicken type (S)	Batch (B)	Number of birds						Total
		Replication ®						
		R_1_	R_2_	R_3_	R_4_	R_5_	R_6_	
S_1_	B_1_	1500	1000	1600	1000	1200	800	7100
	B_2_	1500	1000	1600	1000	1200	800	7100
S_2_	B_1_	1500	1500	2500	2500	–	–	8000
	B_2_	1500	1500	2500	2500	–	–	8000
Total		6000	5000	8200	8200	2400	1600	30,200

S_1_= Broiler chicken, S_2_= *Sonali* chicken, B_1_= Batch 1(Winter: November 2020- February 2021), B_2_= Batch 2 (Summer: March-June 2021).

**Table 2:** Composition of diets for broiler and *Sonali* chickens used in the experiment.

Ingredients	Amount (Kg)			
	Broiler		Sonali	
	Starter diet (1–10 days)	Finisher diet (11–30 days)	Starter diet (1–30 days)	Finisher diet (31–60 days)
Maize	56.50	63.50	52.00	55.50
Soybean meal	25.50	18.50	20.00	20.40
Rice polish	8.00	8.50	13.00	10.00
Protein concentrate	7.50	7.00	7.50	7.40
Limestone	2.00	1.50	3.80	2.50
DCP	–	–	0.50	0.50
Oil	–	0.50	2.50	3.00
Lysine	–	–	0.25	0.25
Methionine	–	–	0.15	0.15
Salt	0.50	0.50	0.30	0.30
Total	100.00	100.00	100.00	100.00
Calculated composition				
ME (kcal/kg)	3035.00	3150.00	2950.00	3000.00
Crude protein (CP) (%)	22.00	21.00	19.50	19.00
Crude fiber (CF) (%)	3.00	3.00	5.50	5.50
Crude fat (%)	4.5.0	5.50	3.50	3.00
Calcium (Ca) (%)	0.90	0.84	0.85	0.85
Phosphorus (P) (%)	0.45	0.42	0.42	0.42
Lysine (%)	1.32	1.19	1.15	1.10
Methionine (%)	0.50	0.48	0.48	0.45

### 2.4 Data recording

The following data were recorded during the investigation.

a **Growth performance**
  i Body weight and feed intake: bi-weekly replication-wise.
  ii Dead birds: when occurred.
  iii FCR (Feed intake/live weight) = $\frac{\;\text{Feed}\;\text{intake}}{\text{Live}\;\text{weight}}$
  iv Production cost (Taka/kg live weight) was calculated considering the chick cost, feed cost, labor cost and vaccine cost, etc.
  v Net profit (Taka/kg live weight) = Price of per kg live bird – production cost of per kg live bird.
b **Meat yield traits**

A total of 20 birds at the end of the experiment, 1 bird in each replicate were taken randomly and then slaughtered and processed (slaughtering, bleeding, scalding, de-feathering, evisceration, washing) to make dressed/ready-to-cook carcass. Thereafter, the dressed carcass was processed as cut-up parts.

The following meat yield data were recorded:

Live weight (g), dressed weight (g), dressing (%), breast meat weight (g), breast meat (%), dark meat weight (g), dark meat (%), thigh weight (g), giblet weight (g).

a **Lipid profiles**

At the end of the experiment, blood samples were collected from the birds during slaughter. Thereafter, the serum of the blood was separated from the blood samples using a centrifuge machine (4000 rpm for 10 min). Then the lipid profiles (total cholesterol, triglyceride, HDL, and LDL) of the samples were measured using a lipid profiles kit (Crescent Diagnostic Lab) by spectrophotometric method.

### 2.5 Statistical analysis

The collected data were analyzed in 2 chicken type × 2 batch factorial design using the Statistix10 computer package program.

#### 2.5.1 Statistical model

The following statistical model was used for the analysis of data.

$$Y_{ijk}=\mu +S_{i}+B_{j}+{\left(S\times B\right)}_{ij}+e_{ijk}$$

Where Y_ijk_ is the observation in the k^th^ replication of the i^th^ chicken type and the j^th^ batch.

µ is the overall mean.

*S*_i_ is the fixed effect of the i^th^ chicken type (*i* = 1, 2).

B_j_ is the fixed effect of the j^th^ batch (*j* = 1, 2).

(*S* × B*)*_ij_ is the interaction effect of the i^th^ chicken type and the j^th^ batch. e_ijk_ is the random error.

## 3 Results and discussion

### 3.1 Growth performances of broiler and *Sonali* chickens

Body weight, feed intake, feed conversion ratio (FCR), and production cost differed significantly between chicken types (Table 3). Broiler chicken had a higher body weight and feed intake than *Sonali* chicken. On the other hand, broiler chicken had a lower FCR and production cost than *Sonali* chicken. Mortality did not differ (*p*>0.05) between broiler and *Sonali* chickens (*p*>0.05). Although there was no significant difference between broiler and *Sonali* chickens for net profit, *Sonali* chickens tended to show a higher net profit than the broiler chickens.

**Table 3:** Growth performance of broiler and *Sonali* chickens at different batches for 30 days and 60 days of age.

Traits	Batch (B)	Chicken type (S)		Mean	LSD value & level of significance+		
		S_1_	S_2_		S	B	SxB
Body weight (g/bird)	B_1_	1549.90	661.50	1105.70	136.050^⁎⁎⁎^	124.200^NS^	157.100^NS^
	B_2_	1590.00	601.00	1095.50			
	Mean	1569.90	631.20	1100.60			
Feed intake (g/bird)	B_1_	2471.10	2148.40	2309.80	382.000*	348.720^NS^	441.100^NS^
	B_2_	2425.50	1875.80	2150.60			
	Mean	2448.30	2012.10	2230.20			
FCR (Feed intake/live weight)	B_1_	1.58	3.07	2.33	0.232^⁎⁎⁎^	0.212^NS^	0.268^NS^
	B_2_	1.52	2.96	2.24			
	Mean	1.55	3.01	2.28			
Mortality (%)	B_1_	2.61	1.27	1.94	1.409^NS^	1.287^NS^	1.627^NS^
	B_2_	1.98	2.09	2.03			
	Mean	2.29	1.68	1.99			
Production cost (Tk/kg live bird)	B_1_	102.84	203.77	153.31	7.995^⁎⁎⁎^	7.299^NS^	9.232^NS^
	B_2_	104.53	199.59	152.06			
	Mean	103.69	201.68	152.68			
							
Net profit (Tk/kg live bird)	B_1_	17.16	16.22	16.392	7.995^NS^	7.299^NS^	9.232^NS^
	B_2_	15.47	20.41	17.942			
	Mean	16.31	18.32	17.317			

+NS, *p*>0.05; *, *p*<0.05; **, *p*< 0.01; ***, *p*< 0.001; NS= Not significance; S_1_= Broiler chicken; S_2_= *Sonali* chicken; B_1_= Batch 1(Winter: November 2020- February 2021); B_2_= Batch 2 (Summer: March-June 2021); Sale (BD Tk/kg live broiler) =120.00; Sale (BD Tk/kg live *Sonali*) = 220.

As for the effect of the batch, no significant difference was observed between broiler and *Sonali* chickens for body weight, feed intake, FCR, production cost, net profit, and mortality. Similarly, no significant difference was found in the interaction of chicken type and batch for the growth performances (*p*>0.05).

In this study, the broiler performed better than the *Sonali* chicken in terms of body weight, feed intake, FCR, and production cost. Khawaja et al. (2013) reported that the body weight gain and FCR of Rural Leghorn (RLH), Fayoumi Rhode Island Red crossbred (FRIR), and Rhode Island Red Fayoumi crossbred (RIRF) crossbreds were 1253.68 g and 4.46, 1230.00 g and 4.55 and 1188.00 g and 4.40, respectively which partially supported the present findings. However, *Sonali* chicken tended to show a higher net profit than broiler chicken because of the preference of consumers and the higher price of *Sonali* chicken. The higher price of chickens depends on the preference of consumers and meat quality. The quality and taste of *Sonali* chicken meat are close to the indigenous chicken meat. Sarker et al. (2008) reported a higher net profit in *Sonali* chicken than in broiler chicken consistent with the present findings. Dutta et al. (2012) reported that the broiler had the highest body weight gain as well as FCR compared to the Fayoumi, ISA Brown and *Sonali* chicken, whereas the highest net profit was in the ISA Brown, followed by *Sonali*, Fayoumi, and broiler chicken, respectively. The net profit in the present study tended to show higher in the *Sonali* chicken compared to the broiler chicken which corroborates with Hannan et al. (2020). They reported the highest net profit in *Sonali* chicken followed by Cockerel and Cobb 500 chickens, respectively. A previous study showed a lower net profit in *Sonali* chickens than in broilers or layers, but higher than in *deshi* chickens (Sarker et al., 2008).

The interaction between chicken type and batch was not significant for the growth performance traits. No previous work was found on the interaction effect of batch and chicken types on their growth performance traits.

### 3.2 Meat yield traits of broiler and *Sonali* chickens

The chicken type was different for live weight, dressed weight, dressing percentage, thigh weight, breast weight, breast meat percentage, dark meat weight, dark meat percentage, and giblet weight (*p*<0.001) (Table. 4). Meat yield traits; live weight, dressed weight, dressing percentage, thigh weight, breast weight, breast meat percentage, dark meat weight, and giblet weight were higher in broiler compared to the *Sonali* chicken, except for dark meat percentage. *Sonali* chicken yielded higher dark meat than broiler chicken.

**Table 4:** Meat yield traits of broiler and *Sonali* chickens at different batches for 30 and 60 days of age.

Traits	Batch (B)	Chicken type (S)		Mean	LSD value & level of significance+		
		S_1_	S_2_		S	B	SxB
Live weight (g/bird)	B_1_	1583.30	916.30	1249.80	220.930^⁎⁎⁎^	220.930^NS^	279.460^NS^
	B_2_	1348.20	990.50	1169.30			
	Mean	1465.80	953.40	1209.60			
Dressed weight (g/bird)	B_1_	965.33	528.00	746.67	158.410^⁎⁎⁎^	158.410^NS^	200.370^NS^
	B_2_	790.17	497.50	643.83			
	Mean	877.75	512.75	695.25			
Dressing (%)	B_1_	60.37	57.86	59.11	4.101*	4.101*	5.187^NS^
	B_2_	58.45	50.51	54.48			
	Mean	59.41	54.18	56.80			
Thigh weight (g)	B_1_	295.17	184.50	239.83	41.692^⁎⁎⁎^	41.692^NS^	52.736^NS^
	B_2_	246.17	185.00	215.58			
	Mean	270.67	184.75	227.71			
Breast weight (g)	B_1_	273.67	86.75	180.21	57.270^⁎⁎⁎^	57.270^NS^	72.441^NS^
	B_2_	221.17	88.00	154.58			
	Mean	247.42	87.38	167.40			
Breast meat (%)	B_1_	27.87	16.67	22.27	2.439^⁎⁎⁎^	2.439^NS^	3.086^NS^
	B_2_	27.65	17.70	22.68			
	Mean	27.76	17.19	22.48			
Dark meat weight (g)	B_1_	691.67	441.25	566.46	105.440^⁎⁎⁎^	105.440^NS^	133.370^NS^
	B_2_	569.00	409.50	489.25			
	Mean	630.33	425.37	527.85			
Dark meat (%)	B_1_	72.12	83.32	77.73	2.439^⁎⁎⁎^	2.439^NS^	3.085^NS^
	B_2_	72.34	82.28	77.31			
	Mean	72.24	82.80	77.52			
Giblet weight (g)	B_1_	135.83	64.95	100.39	7.891^⁎⁎⁎^	7.891^NS^	9.982^NS^
	B_2_	131.83	66.29	99.06			
	Mean	133.83	65.62	99.73			

+NS, *p*>0.05; *, *p*<0.05; **, *p*< 0.01; ***, *p*< 0.001; NS= Not significance; S_1_= Broiler chicken; S_2_= *Sonali* chicken; B_1_= Batch 1(Winter: November 2020- February 2021); B_2_= Batch 2 (Summer: March-June 2021).

The batch was not different for live weight, dressed weight, dressing percentage, thigh weight, breast weight, breast meat percentage, dark meat weight, and giblet weight (*p*>0.05), except for dressing percentage (*p*<0.05). The B_1_ had a higher dressing percentage than B_2_.

The interaction between chicken type and batch was not significant for meat yield traits (*p*>0.05).

In the present study, broiler chicken performed better than *Sonali* chicken in terms of live weight, dressed weight, dressing percentage, thigh weight, breast weight, breast meat percentage, dark meat weight and giblet weight. However, broiler chicken had lower dark meat percentage (*p*>0.05) than *Sonali* chickens. The previous findings showed the highest edible meat in the cockerel, moderate in the broiler, *Sonali,* and indigenous chickens, and the lowest in RIR chicken which partially supported the present findings (Islam & Dutta, 2010). On the other hand, Khawaja et al. (2013) reported the highest dressing percentage in Fayoumi male_ Rhode Island Red female (FRIR) moderate in reciprocal F_1_ crossbred of RIR_Fayoumi (RIRF) and the lowest in White Leghorn male_F_1_ female (RLH) which was also partially supported the present findings.

There was no literature of previous work found on the effect of the batch and the interaction of chicken type and batch on meat yield traits of broiler and *Sonali* chickens.

### 3.3 Lipid profiles of broiler and *Sonali* chicken

The chicken type was not different for total cholesterol, triglyceride, high-density lipoprotein, and low-density lipoprotein (*p*>0.05) (Table. 5). However, broiler tended to increase total cholesterol, triglyceride, and high-density lipoprotein compared to *Sonali* chickens.

**Table 5:** Lipid profiles of broiler and *Sonali* chickens at different batches for 30 days and 60 days of age.

Traits	Batch (B)	Chicken type (S)		Mean	LSD value & level of significance+		
		S_1_	S_2_		S	B	SxB
Cholesterol (mg/dl)	B_1_	153.30	153.74	153.52	15.696^NS^	15.696^NS^	19.854^NS^
	B_2_	156.15	135.56	145.86			
	Mean	154.72	144.65	149.69			
TG (mg/dl)	B_1_	181.34	160.60	170.97	20.833^NS^	20.833^NS^	26.352^NS^
	B_2_	164.86	155.98	160.42			
	Mean	173.10	158.29	165.69			
HDL (mg/dl)	B_1_	48.96	45.21	47.08	16.669^NS^	16.669^NS^	21.085^NS^
	B_2_	53.75	38.50	46.12			
	Mean	51.35	41.85	46.60			
LDL (mg/dl)	B_1_	68.08	76.41	72.24	26.080^NS^	26.080^NS^	32.988^NS^
	B_2_	69.43	65.87	67.65			
	Mean	68.75	71.14	69.95			

+NS, *p*>0.05; *, *p*<0.05; **, *p*< 0.01; ***, *p*< 0.001; NS= Not significance; S_1_= Broiler chicken; S_2_= *Sonali* chicken; B_1_= Batch 1(Winter: November 2020-February 2021); B_2_= Batch 2 (Summer: March-June 2021); TG= Triglyceride; HDL= High-density lipoprotein; LDL= Low-density lipoprotein.

As to the effect of the batch, there was no significant difference between batches for total cholesterol, triglyceride, high-density lipoprotein, and low-density lipoprotein (*p*>0.05). Lipid profiles did not differ between B_1_ and B_2_.

The chicken type did not interact with the batch for total cholesterol, triglyceride, high-density lipoprotein and low-density lipoprotein.

Chicken types did not differ for lipid profiles (total cholesterol, triglyceride, high-density lipoprotein, and low-density lipoprotein (*p*>0.05). However, broiler chickens tended to increase lipid profiles, except for low-density lipoprotein compared with *Sonali* chickens. The present findings contradicted the findings of Dutta et al. (2013). They estimated the highest amount of cholesterol in RIR, followed by *Sonali*, Cobb 500, Cockerel, Fayoumi, and Indigenous chickens, respectively.

No previous work was found on the effect of the batch, and the interaction between chicken type and batch on the lipid profile content of the blood of broiler and Sonali chickens.

## 4 Conclusions

The present study reveals that broiler chicken performed better than *Sonali* chicken in terms of growth performance and meat yield traits. The chicken type did not affect lipid profiles (total cholesterol, Triglyceride, HDL, and LDL) content in the blood of chickens and net profit. However, Sonali chicken tended to show higher net profit and lower total cholesterol and triglyceride compared to broiler chicken. As to the effect of batch (B), batches (B_1_ and B_2_) did not affect growth performance, meat yield, and lipid profile content in the blood of chickens. No interaction between chicken type and the batch was found in growth performance, meat yield traits, and lipid profile content in the blood of chickens. Therefore, *Sonali* chicken may be suitable for producing consumer preferable and profitable chicken meat. However, more studies are needed to confirm the present findings and suggest to use of a suitable chicken type for commercial purposes.

## Funding

This research received no specific grant from funding agencies in the public, commercial, or private sectors.

## Ethical statement

The study was approved by the Institutional Committee on Animal Care and Use in Research (ICACUR) of Bangabandhu Sheikh Mujibur Rahman Agricultural University (No. BSMRAU/DEAN/FVMAS/25/ICACUR/19).

## Declaration of Competing Interest

No potential conflicts of interest to declare
